# On the Use of Quinine in the Treatment of Ulcers

**Published:** 1853-11

**Authors:** L. N. Coon

**Affiliations:** Warrenville


ART. II.—*On the Use of Quinine in the Treatment of Ulcers.* By L. N. COON, M. D.

I AM induced to write this short article, however imperfect it may be, for the purpose of adding my testimony in favor of the beneficial effects of the *Sulphate of Quinine* as a topical agent in the treatment of sloughing, indolent ulcers, as set forth by A. J. Wedderburn, M. D., Professor of Anatomy in the University of Louisiana.

Surely too much cannot be said in praise of an agent, whose application ( judging from my limited experience) bids so fair to prove one of the first remedies in the Materia Medica, for the purpose above mentioned. I doubt not that others have found it equally effectual, in removing that peculiar indolent condition to which they are so frequently inclined.

If it is the duty of medical men to investigate and study the nature and applicability of various remedies, it is true also that their suggestions should be regarded by those who are not willing to investigate for themselves, or those who are not competent to this task. These should be ready to put into practice (on all suitable occasions) and carry out the suggestions of their superiors. By thus carefully and candidly applying and watching their effects in given cases, and under varying circumstances, we may be the humble instruments of establishing, or assisting to establish beyond a doubt, a correct and reliable course of practice in a great variety of cases.

I know that there is a disposition on the part of physicians, to some extent, to neglect the sayings of others, from the fact that they themselves were not the first to make the discovery, or perhaps more correctly speaking, the cause is a *profound indifference* to the wants, necessities, honor, and real advancement of the medical profession. But perhaps I am not justifiable in thus finding fault I trust I shall be excused, while I give a brief statement of a case which came under my notice and care, sometime in November last.

T. M., aged 28 years, of rather strong constitution, and temperate habits. Present condition of patient—great emaciation, his countenance presented that of marked *anæma*; was able to sit up only as supported by an attendant; pulse small and soft, rather quick; slight fever, an occasional flush on the cheek; slight cough; bowels loose. Condition of leg—ulcers extending from the patella two-thirds of the distance to the groin, covering over nearly the whole anterior portion of the thigh, spreading, breaking down and involving new tissue in the general rim. This ulcerated condition extending below the knee something like six inches, the whole surface above and below the patella presenting a number of sinuous openings, discharging a sinuous-like fluid. These ulcers presented a dark colored appearance around them, some of them were formed by the flesh sloughing out. A probe would pass in any direction from one opening to another.

*Treatment.*—I first ordered the poultices which were then on, and had been for three weeks past, to be removed, then ordered the part to be washed with castile soap and rain-water. I then injected with a syringe a solution of Sulphate of Quinine, 10 grs. to 1 oz. of water, the whole surface was then covered with equal parts of quinine and flour, gentle compression was used by a light roller. He was then ordered as a constitutional remedy, a syrup composed of Sarsaparilla and Yellowdock. This treatment without variation was continued for two or three weeks, during which time the patient rapidly improved, until he was discharged perfectly sound, and in good health, with the exception of some stiffness of the joint.

Now, I am satisfied that no other form of treatment, could have so rapidly and effectually cured this patient, as the one adopted. I attribute it all, or nearly so, to the stimulating effects of the quinine, which seemed to give to the ulcers a new and healthy action, and to change the color and appearance of the sores, within forty-eight hours after their application. I hope that others will be induced to give this agent a fair trial, and communicate to us their experience.

Warrenville, Aug. 30. 1835.

